# Spasmolytic Mechanism of Aqueous Licorice Extract on Oxytocin-Induced Uterine Contraction through Inhibiting the Phosphorylation of Heat Shock Protein 27

**DOI:** 10.3390/molecules22091392

**Published:** 2017-08-29

**Authors:** Lu Yang, Cheng-Zhi Chai, Yan Yan, Ying-Dan Duan, Astrid Henz, Bo-Li Zhang, Anders Backlund, Bo-Yang Yu

**Affiliations:** 1 Department of Complex Prescription of TCM, Jiangsu Provincial Key Laboratory for TCM Evaluation and Translational Research, China Pharmaceutical University, 639 Longmian Road, Nanjing 211198, China; lu.yang@stu.cpu.edu.cn (L.Y.); chaichengzhi@126.com (C.-Z.C.); yingdanduan@stu.cpu.edu.cn (Y.-D.D.); 2 Divsion of Pharmacognosy, Department of Medicinal Chemistry, Uppsala University, BMC box 574, S-751 23 Uppsala, Sweden; astrid.henz@fkog.uu.se; 3 Modern Research Center for Traditional Chinese Medicine, Shanxi University, No. 92, Wucheng Road, Taiyuan 030006, China; yanyan520@sxu.edu.cn; 4 Institute of Traditional Chinese Medicine, Tianjin University of Traditional Chinese Medicine, Tianjin 300193, China

**Keywords:** licorice, uterine contraction, HSP27 phosphorylation, ChemGPS-NP prediction, molecular docking

## Abstract

Licorice derived from the roots and rhizomes of *Glycyrrhiza uralensis Fisch*. (Fabaceae), is one of the most widely-used traditional herbal medicines in China. It has been reported to possess significant analgesic activity for treating spastic pain. The aim of this study is to investigate the spasmolytic molecular mechanism of licorice on oxytocin-induced uterine contractions and predict the relevant bioactive constituents in the aqueous extract. The aqueous extraction from licorice inhibited the amplitude and frequency of uterine contraction in a concentration-dependent manner. A morphological examination showed that myometrial smooth muscle cells of oxytocin-stimulated group were oval-shaped and arranged irregularly, while those with a single centrally located nucleus of control and licorice-treated groups were fusiform and arranged orderly. The percentage of phosphorylation of HSP27 at Ser-15 residue increased up to 50.33% at 60 min after oxytocin stimulation. Furthermore, this increase was significantly suppressed by licorice treatment at the concentration of 0.2 and 0.4 mg/mL. Colocalization between HSP27 and α-SMA was observed in the myometrial tissues, especially along the actin bundles in the oxytocin-stimulated group. On the contrary, the colocalization was no longer shown after treatment with licorice. Additionally, employing ChemGPS-NP provided support for a preliminary assignment of liquiritigenin and isoliquiritigenin as protein kinase C (PKC) inhibitors in addition to liquiritigenin, isoliquiritigenin, liquiritin and isoliquiritin as MAPK-activated protein kinase 2 (MK2) inhibitors. These assigned compounds were docked with corresponding crystal structures of respective proteins with negative and low binding energy, which indicated a high affinity and tight binding capacity for the active site of the kinases. These results suggest that licorice exerts its spasmolytic effect through inhibiting the phosphorylation of HSP27 to alter the interaction between HSP27 and actin. Furthermore, our results provide support for the prediction that potential bioactive constituents from aqueous licorice extract inhibit the relevant up-stream kinases that phosphorylate HSP27.

## 1. Introduction

Uterine contractions with low frequency and amplitude occurs throughout the entire menstrual cycle. However, the amplitude increases dramatically at the time of menstruation for patients with primary dysmenorrhea (PD), which subsequently has a significant negative impact on their study, work and normal lives [1]. Moreover, severe uterine contractions during premature labor even results in a high risk of newborn deaths [2,3]. Consequently, the relaxation of uterine spasm is a crucial strategy for the treatment of PD and premature labor.

Heat shock protein 27 (HSP27), a member of the small heat shock proteins family with a low-molecular weight, is a 15–30 kDa protein encoded by the *HSPB1* gene and typically exists as large, polydisperse assemblies [4,5]. HSP27 appears in all types of muscle cells with multiple functions of ATP-independent chaperone activity, thermo-tolerance, regulation of cell differentiation, cell migration, apoptosis, membrane stability, actin polymerization and muscle contraction [6]. Recently, it has been put forward that the phosphorylation of HSP27 is associated with the change of quaternary structure and plays an important role in regulation of uterine contractions. It has been regarded as a potential target for the relevant diseases such as labor and PD [3].

Licorice is derived from the roots and rhizomes of *Glycyrrhiza uralensis Fisch*. (Fabaceae), a leguminous perennial herb. It is a commonly used Chinese herbal medicine with the main indications of detoxification, relieving cough and reducing sputum, relieving sore-throat, hepatoprotection, analgesia, etc. [7,8,9,10]. The extracts used from *Glycyrrhiza uralensis* have a complex chemical composition and more than 400 compounds have been identified from Glycyrrhiza species, including flavonoids, triterpenoid saponins, coumarin and chalcones [11]. The major compounds, such as glycyrrhizin, liquiritin, liquiritigenin and isoliquiritigenin, have been reported to exert a variety of biological activities including being anti-inflammatory, antidiabetic, antibacterial, antioxidant, anticancer and antispasmodic [12,13]. Jia et al. demonstrated that an aqueous licorice extract exerted spasmolytic effects on isolated mouse uteri, of which contractions were aroused by various stimulants, including potassium chloride, acetylcholine, carbachol, oxytocin or bradykinin. However, little is known about its molecular mechanism and bioactive constituents [14,15].

In the present study, the spasmolytic efficacy of aqueous licorice extract was monitored by a force-displacement transducer on an ex vivo model of oxytocin-induced uterine contraction. Simultaneously, the morphological change of myometrial smooth muscle cells was performed by histological examination. The phosphorylation of heat shock protein 27 (HSP27) was studied to explore the underlying molecular mechanism of licorice on spasmolysis. Moreover, UHPLC-Q Extractive Orbitrap-HRMS analysis was used to identify the seven principal chemical constituents in the licorice aqueous extract. ChemGPS-NP, a tool for navigating the chemical property space of natural products, was used to predict the biological activities of the constituents in the licorice aqueous solution [16,17]. Subsequently, the potential of the predicted bioactive ingredients as relevant up-stream kinase inhibitors was scrutinized by molecular docking.

## 2. Results

### 2.1. UHPLC-Q Extractive Orbitrap-HRMS Chromatograms of Licorice Aqueous Extract

**Figure 1:**
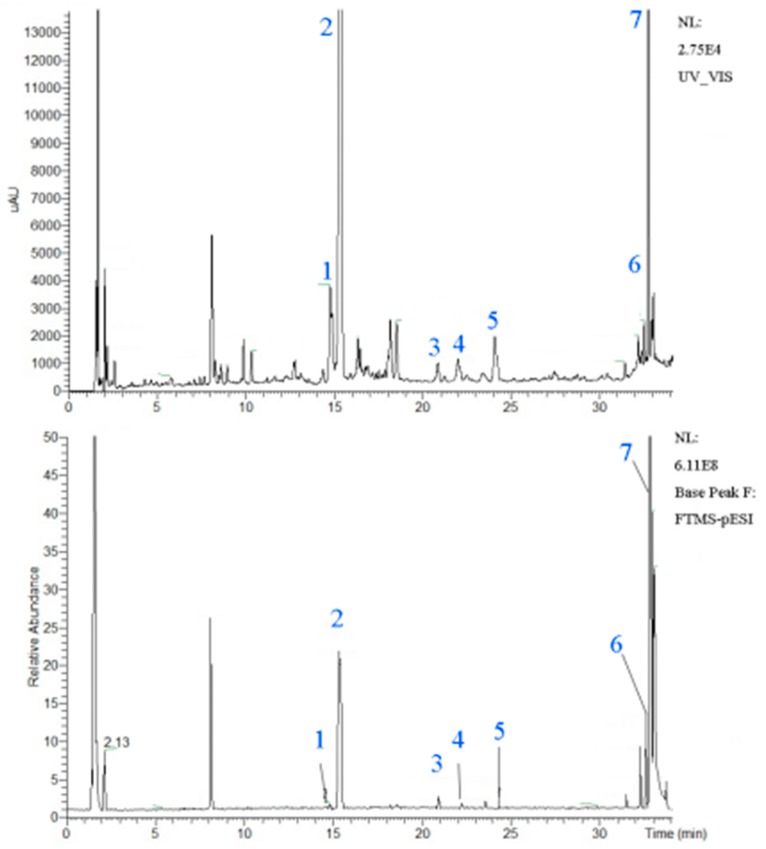
UHPLC-Q Extractive Orbitrap-HRMS Chromatograms of the licorice aqueous extract. The seven peaks correspond to (**1**) liquiritin apioside or isomer; (**2**) liquiritin; (**3**) isoliquiritin apioside; (**4**) isoliquiritin; (**5**) liquiritigenin; (**6**) isoliquiritigenin and (**7**) glycyrrhetinic acid.

The typical chromatogram of the licorice aqueous extract is shown in Figure 1, while characterization of chemical constituents of licorice aqueous extracts by UHPLC-Q Extractive Orbitrap-HRMS is exhibited in Table 1. There are seven peaks, which correspond to (**1**) liquiritin apioside or isomers; (**2**) liquiritin; (**3**) isoliquiritin apioside; (**4**) isoliquiritin; (**5**) liquiritigenin; (**6**) isoliquiritigenin and (**7**) glycyrrhetinic acid. The data from HPLC quantification are shown in Table 2 and the values are expressed as mean ± SD (*n* = 6, 6 batches of preparations). Glycyrrhetinic acid and liquiritin were identified as the major compounds, which had a mass of 21.60 mg/g and 11.82 mg/g respectively.

### 2.2. Effects of Licorice Aqueous Extract on Oxytocin-Stimulated Uterine Contraction Ex Vivo

**Figure 2:**
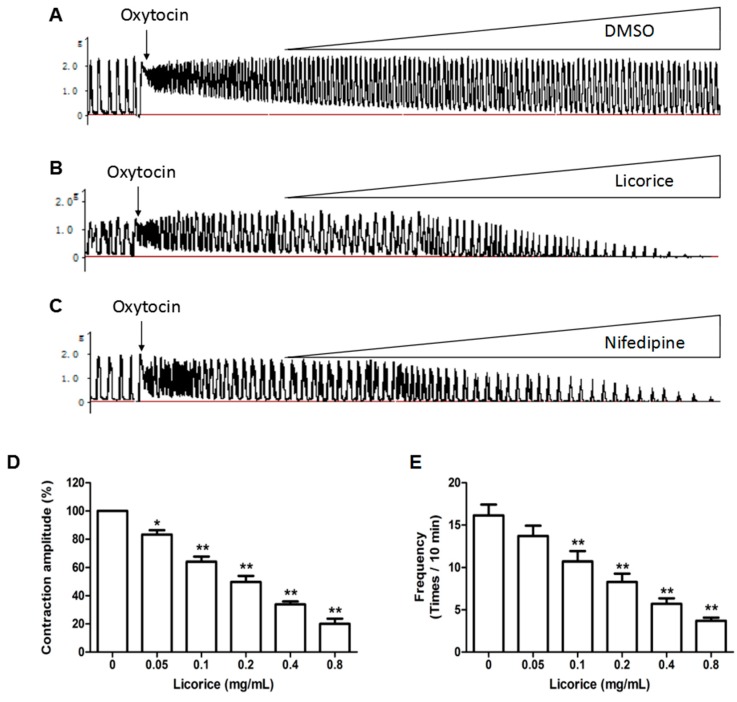
Effects of licorice aqueous extract on oxytocin-stimulated uterine contraction ex vivo: (**A**) Original tracing example of contractions stimulated by oxytocin (0.01 U/mL) in isolated mouse uterus with DMSO treatment (0.02–0.2%); (**B**) Original tracing example of relaxant effects of licorice aqueous extract (0.05–0.8 mg/mL) on oxytocin-stimulated contractions; (**C**) Original tracing example of relaxant effects of nifedipine (positive control, 0.125–1 ng/mL) on oxytocin-stimulated contractions; (**D**) Concentration-dependent effects of licorice on the contraction amplitude; (**E**) Concentration-dependent effects of licorice on the contraction frequency. Each column represents the mean ± SD (*n* = 6). * = *p* < 0.05, ** = *p* < 0.01 for the licorice-treated group vs. oxytocin-stimulated group. Statistical significance was analyzed by using one-way ANOVA followed by Tukey’s Honestly Significant Difference test.

The licorice aqueous extract exerted an inhibitory effect on the oxytocin-induced uterine contractions in a concentration-dependent manner at a concentration of 0.05–0.8 mg/mL. Figure 2A–C shown the typical traces recorded from the uterine strips. Uterine strips were treated with DMSO, licorice and nifedipine separately. Figure 2D,E summarized the significant inhibition of licorice on uterine contraction amplitude and frequency.

### 2.3. Effects of Licorice Aqueous Extract on Morphological Changes in the Uterine Inner Annular Layer

**Figure 3:**
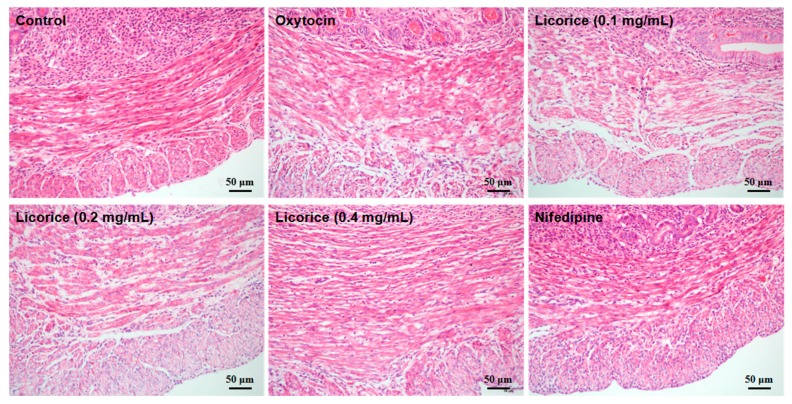
Effects of licorice aqueous extract on morphological changes in the uterine inner annular layer. Example histological section of the uterine inner annular layer using HE staining under the light microscope at 200× objective. No discernible pathological changes were observed in the control group and the myometrial cells were slender with round nuclei, which was described as fusiform nucleated cells. The short rod-shaped cells with shorter nuclei were in contractile status, which was described as oval nucleated cells. The licorice aqueous extract downregulated the proportion of oval nucleated cells (contractile cells) in uterine inner annular layer.

As shown in Figure 3, the morphological examination of uterine inner annular layers showed that the myometrial smooth muscle cells were characterized by an oval shape and arranged irregularly (contractile cells) in the oxytocin-stimulated model group when compared with the fusiform nucleated cells of the control group. In the licorice-treated groups and nifedipine group, the myometrial cells were fusiform and arranged orderly with a single centrally located nucleus. These figures show that the licorice aqueous extract downregulated the proportion of oval-nucleated cells (contractile cells) in the uterine inner annular layer.

### 2.4. Effects of Licorice Aqueous Extract on Phosphorylated HSP27 Expression in Oxytocin-Stimulated Uterus

**Figure 4:**
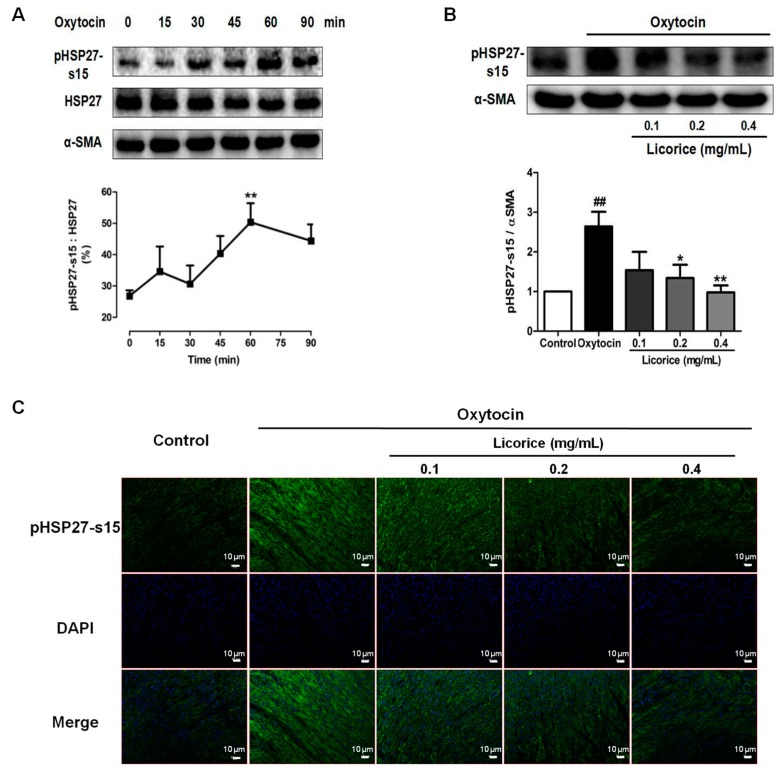
Effects of licorice aqueous extract on phosphorylated HSP27 expression in oxytocin-stimulated uterus. (**A**) Example of a Western blot analysis of phosphorylated HSP27 (p-HSP27-s15), total HSP27 and α-SMA proteins in uterus at six time points after oxytocin stimulation. Data are presented as the mean ± SD (*n* = 3) for each time point. ** = *p* < 0.01 for the oxytocin-stimulated group at 60 min vs. oxytocin-stimulated group at 0 min. Statistical significance was analyzed by using one-way ANOVA followed by Tukey’s Honestly Significant Difference test; (**B**) Example of a Western blot analysis of phosphorylated HSP27 (p-HSP27-s15) proteins with an internal control of α-SMA in uterus of each treatment group. Data illustrated on the graph bar represent the mean ± SD (*n* = 3). ## = *p* < 0.01 for the oxytocin-stimulated model group vs. control group; * = *p* < 0.05 and ** = *p* < 0.01 for the licorice-treated group vs. oxytocin-stimulated group. Statistical significance was analyzed by using one-way ANOVA followed by Tukey’s Honestly Significant Difference test; (**C**) Representative immunofluorescence microscope images of p-HSP27-s15 (green) with DAPI-stained nuclei (blue) in the myometrial tissues were generated using Leica Microsystems (Leica DM2500, Wetzlar, Germany) with a scale bar of 10 μM (*n* = 3).

In order to determine the inhibitory effect of licorice on oxytocin-induced phosphorylation of HSP27, a Western blot analysis was performed. As seen in Figure 4A, oxytocin promoted the phosphorylation of HSP27 at the Ser-15 residue with no effect on the total HSP27 levels and the percentage of p-HSP27-s15 increased up to 50.33% at 60 min after oxytocin stimulation. Licorice aqueous extract significantly suppressed the increased level of phosphorylated HSP27 at the concentration of 0.2 and 0.4 mg/mL (Figure 4B,C).

### 2.5. Effects of Licorice Aqueous Extract on the Colocalization of HSP27 with α-SMA in Oxytocin-Stimulated Uterus

**Figure 5:**
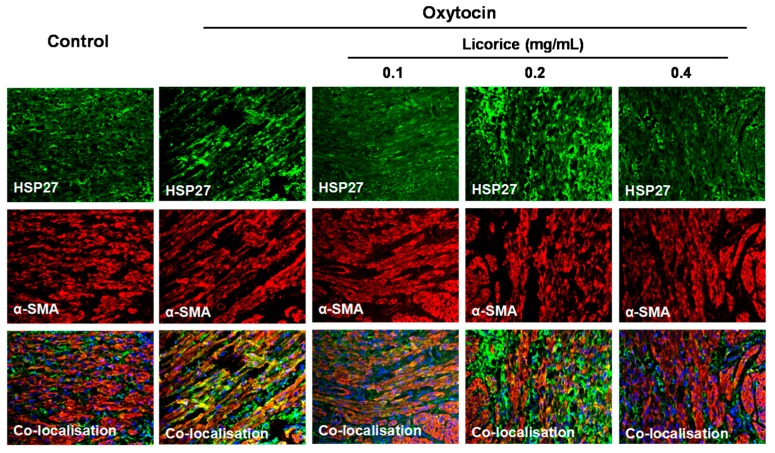
Effects of licorice aqueous extract on the colocalization of HSP27 with α-SMA in oxytocin-stimulated uterus. Representative immunofluorescence microscope images of HSP27 (green), α-SMA (red) and colocalization regions (yellow) with DAPI-stained nuclei (blue) in the myometrial tissues were generated using Leica Microsystems (Leica DM2500, Wetzlar, Germany) with a scale bar of 20 μM (*n* = 3).

In the control group, low levels of colocalization between HSP27 and α-SMA were observed. In contrast, the oxytocin-stimulated model group displayed high levels of colocalization between HSP27 and α-SMA in the myometrial tissues, particularly along actin bundles. This colocalization was lost after the treatment of licorice (Figure 5).

### 2.6. ChemGPS-NP Analysis of Seven Chemical Ingredients from Licorice Aqueous Extract

**Figure 6:**
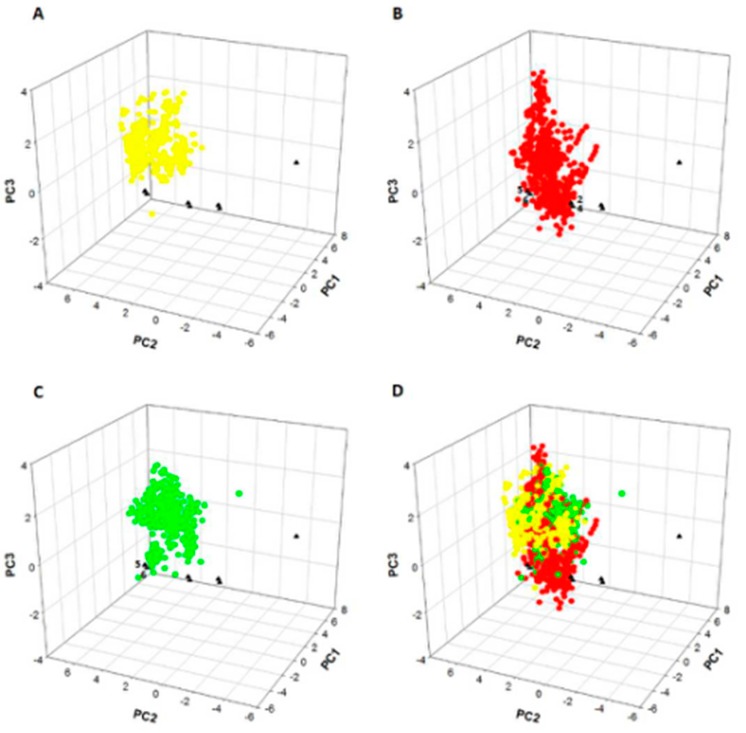
ChemGPS-NP analysis of seven chemical ingredients from the licorice aqueous extract. Score plot of the three dimensions consisting of PC1, PC2 and PC3 from analysis of seven ingredients from licorice aqueous extract: (**1**) liquiritin apioside; (**2**) liquiritin; (**3**) isoliquiritin apioside; (**4**) isoliquiritin; (**5**) liquiritigenin; (**6**) isoliquiritigenin and (**7**) glycyrrhetinic acid as black triangles with a reference set of known inhibitors targeting p38 (yellow), MK2 (red) and PKC (green) in the ChemGPS-NP model. (**A**) The seven chemical ingredients (black triangles) were positioned in the resulting map of known p38 inhibitors (yellow); (**B**) The seven chemical ingredients (black triangles) were positioned in the resulting map of known MK2 inhibitors (red); (**C**) The seven chemical ingredients (black triangles) were positioned in the resulting map of known PKC inhibitors (green); (**D**) The seven chemical ingredients (black triangles) were positioned in the resulting map of known p38 (yellow), MK2 (red) and PKC (green) inhibitors.

ChemGPS-NP is a global chemical space map based on the principal component analysis of physico-chemical properties, which are calculated directly from structure information of natural products. This map is used for the exploration and prediction of the regions with biologically relevant functions and activities. By employing a score prediction in the ChemGPS-NP model, the known inhibitors targeting PKC (green), p38 (yellow) and MK2 (red) were set as reference compounds. As shown in Figure 6, the seven chemical ingredients (black triangles) from the licorice aqueous extract were positioned in the resulting maps. It can be concluded that these seven test compounds do not unambiguously belong to defined group of p38 inhibitors and provided the support for preliminary classifications of liquiritigenin and isoliquiritigenin (Compd. **5** and **6**) as PKC inhibitors in addition to liquiritin, isoliquiritin, liquiritigenin and isoliquiritigenin (Compd. **2**, **4**, **5** and **6**) as MK2 inhibitors, which indicated that their possible activities were analogous to the reference compounds.

### 2.7. Molecular Docking

**Figure 7:**
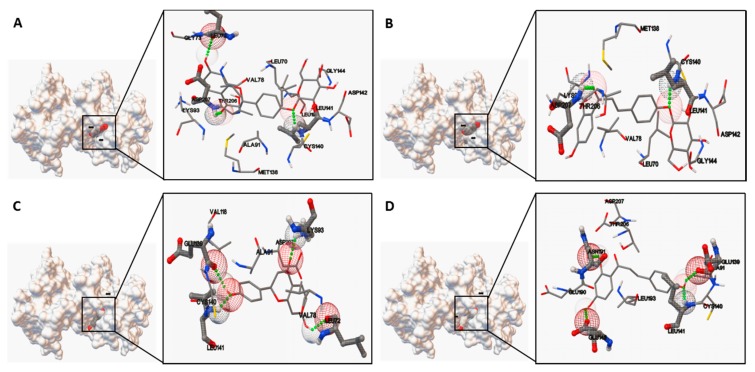
Ligand interaction and comparative binding sites diagrams of the potent ATP competitive inhibitors of MK2: (**A**) liquiritin; (**B**) isoliquiritin; (**C**) liquiritigenin and (**D**) isoliquiritigenin.

**Figure 8:**
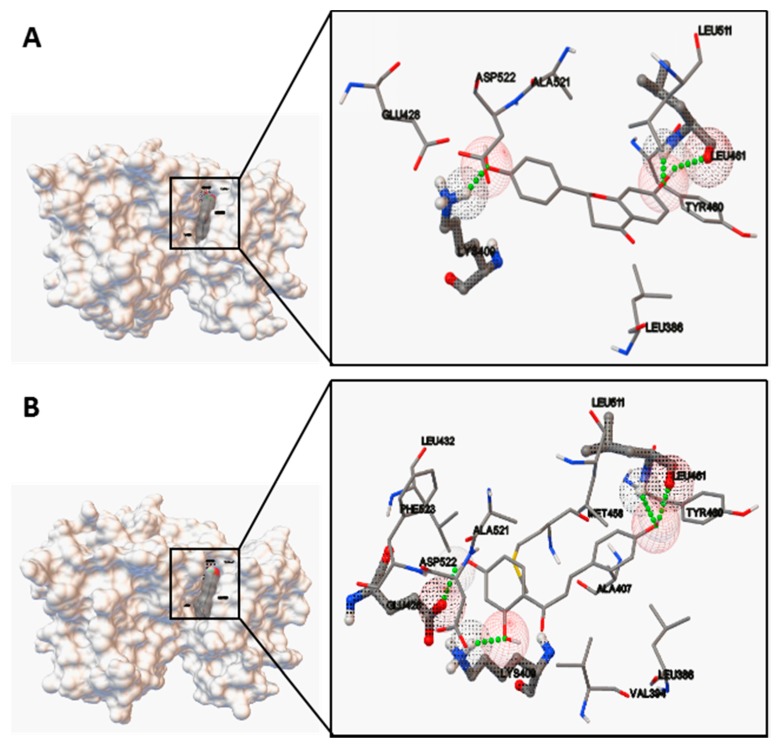
Ligand interaction and comparative binding sites diagrams of (**A**) liquiritigenin and (**B**) isoliquiritigenin in the active site of catalytic domain of PKC.

Based on molecular docking studies, liquiritin, isoliquiritin, liquiritigenin and isoliquiritigenin were docked using Autodock to evaluate the binding site-directed inhibition of MK2. Complexes of MK2 with a potent ATP competitive inhibitor were formed in a stable manner, which was located in the binding pocket of MK2 (Figure 7). As summarized in Table 3, the binding energies were −7.65, −6.69, −6.97 and −6.74 kcal/mol respectively. The hydrogen bonds formed at the active site residues, which indicated the high affinity and tight binding capacity to the kinase MK2. Additionally, the catalytic groove of PKC was occupied by liquiritigenin and isoliquiritigenin with a binding energy of −7.15 and −6.61 kcal/mol, respectively (Figure 8). In the case of liquiritigenin, three hydrogen bonds formed at the amino acid LEU461 and LYS409 of the PKC protein. Isoliquiritigenin also formed stable complex with four hydrogen bonds at the residues of LEU461, GLU426 and LYS409 (Table 4).

## 3. Discussion

Uterine hypercontractility induces the painful menstrual cramps of adolescents and even results in a high risk of newborn deaths during premature labor. Licorice is one of the most commonly-used Chinese herbal medicine and has a long history of treating spastic pain. The present study showed that the aqueous extract from licorice significantly inhibited the amplitude and frequency of oxytocin-stimulated uterine contractions in a concentration-dependent manner. From the morphological examination, licorice obviously maintained the fusiform-shaped morphology of myometrial smooth muscle cells with regular arrangement and decreased the proportion of contractile cells in uterine inner annular layers, which was consistent with the functional evaluation.

HSP27 is a uterine contraction-related protein. These proteins can directly interact with each other to form homo-oligomers or hetero-oligomeric complexes to modulate actin filament formation, dynamics and contraction. These quaternary structures are tightly associated with its function and can be regulated by phosphorylation [18,19]. Actually, the phosphorylation of HSP27 can result in the destabilization and dissociation of the HSP27 oligomer, shifting the equilibrium into small monomer or dimer subunits. Furthermore, this can induce the alteration of the tertiary structure to change its interaction with the actin cytoskeleton [20,21,22]. Eventually, the phosphorylation of HSP27 on serine residue 15 increases its stability and propensity to bind to actin filaments, which can promote uterine contraction and is involved in various human diseases such as labor and primary dysmenorrhea [2,3]. In the present study, the percentage of phosphorylation of HSP27 at Ser-15 residue increased up to 50.33% at 60 min after oxytocin stimulation and the increase was significantly suppressed by licorice. Colocalization between HSP27 and α-SMA was observed in the myometrial tissues, especially along the actin bundles in the oxytocin-stimulated model group. On the contrary, it was no longer colocalized after treatment with licorice. These results demonstrated that licorice can exert spasmolytic effects through inhibiting the phosphorylation of HSP27 to alter the interaction between HSP27 and actin in addition to probably promoting the non-phosphorylated HSP27 to sequester actin monomers, thus decreasing actin polymerization.

In vivo, it has been proven that HSP27 can be phosphorylated at the serine residue 15 by protein kinase C (PKC), p38 mitogen-activated protein kinases (p38 MAPK) and MAP kinase-activated protein kinase 2 (MK2) [23]. The signal transduction cascades involving p38 MAPK and MK2-regulated phosphorylation of HSP27 are important in modulating filament dynamics [21]. PKCδ, the most efficient kinase in phosphorylating HSP27, indirectly phosphorylates HSP27 through activation of the p38 MAPK/MK2 pathway, which has been confirmed by Takai et al. [24]. ChemGPS-NP is a global chemical space map based on physico-chemical properties, which is used for predicting the analogous and biologically relevant activities of the natural products [16,17]. This map can be economically and efficiently used for investigating the bioactive constituents from Chinese medicine characterized by multiple components, targets and pathways [25]. In the present study, ChemGPS-NP was used to predict the specific bioactive constituents targeting the above-mentioned kinases responsible for the phosphorylation of HSP27 from the aqueous extract of licorice. As the results showed, liquiritigenin and isoliquiritigenin were preliminarily classified as PKCδ inhibitors while liquiritin, isoliquiritin, liquiritigenin and isoliquiritigenin as MK2 inhibitors depending on the similar physico-chemical properties referring to known kinase inhibitors. This predicted information explained the bioactive constituents of licorice extract responsible for the particular pharmacological mechanism regulating the phosphorylation of HSP27.

Moreover, the predicted bioactive ingredients were scrutinized by molecular docking as kinase inhibitors. The results showed each complex of the MK2 or PKC catalytic domain with the corresponding compound had a stable formation with low binding energy (Figure 7 and Figure 8). The interaction of hydrogen bonds at the specific active site residues (Table 3 and Table 4) indicated the high affinity and tight binding capacity as the relevant kinase inhibitors.

**Figure 9:**
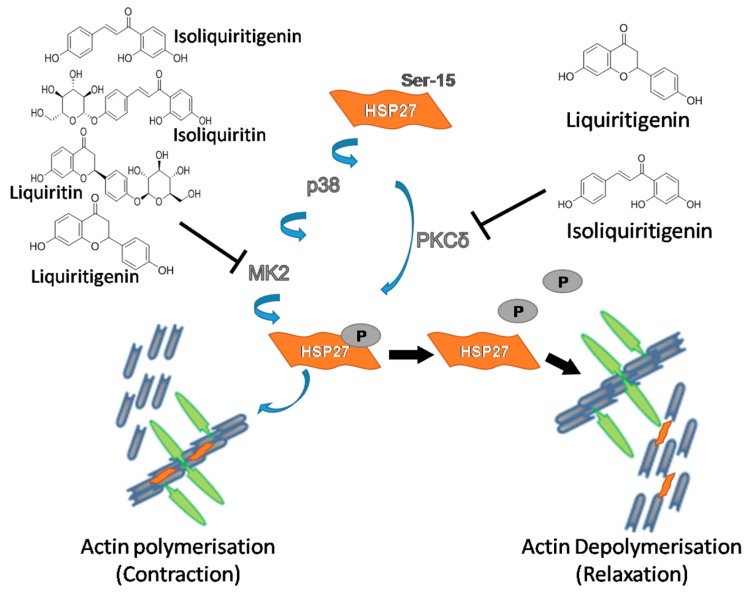
Schematic representation of a model for spasmolytic mechanism of bioactive constituents from aqueous licorice extract on uterine contraction through inhibiting the phosphorylation of heat shock protein 27.

In conclusion, our results revealed that licorice exerted a uterine relaxant effect through inhibiting the phosphorylation of HSP27 to alter the interaction of HSP27 and actin, which was due to the possible inhibitory effect of particular bioactive constituents on the up-stream kinase of MK2 or PKC regulating the phosphorylation of HSP27 (Figure 9).

## 4. Materials and Methods

### 4.1. Materials and Reagents

The licorice was purchased from Nanjing Traditional Chinese Medicine Out-patient Department (Nanjing, Jiangsu, China) and verified as un-compromised authentic crude drugs by the corresponding author Boyang Yu. Estradiol benzoate for injection was obtained from Tianjin Jinyao Amino Acid Pharmaceutical Co., Ltd. (Tianjin, China). Oxytocin was purchased from Nanjing Xinbai Pharmaceutical Co., Ltd. (Nanjing, Jiangsu, China). The HSP27, pHSP27-Ser15 and α-SMA primary antibodies, Alexa Fluor^®^ 647 conjugated anti-rabbit and Alexa Fluor^®^ 488 conjugated anti-mouse secondary antibodies were obtained from Abcam (Burlingame, CA, USA). Anti-fade mounting reagent, DAPI staining solution, radioimmunoprecipitation assay buffer (RIPA), protein assay kit (BCA), phosphate-buffered saline (PBS) and bovine serum albumin (BSA) were obtained from Beyotime Institute of Biotechnology (Shanghai, China). Liquiritin, isoliquiritin, liquiritigenin, isoliquiritigenin and glycyrrhizic acid were purchased from Chunqiu Bio-Technology Co., Ltd. (Nanjing, Jiangsu, China).

### 4.2. Preparation of Licorice Aqueous Extract and UHPLC-Q Extractive Orbitrap-HRMS Analysis

Licorice crude drug was soaked in ten-fold ddH2O (1:10, *w*/*v*) for 0.5 h, before being decocted for 1 h. Subsequently, the extraction was filtered through six layers of gauze, before the residue was boiled twice in a total volume of 8 and 5 times (*w*/*v*) the weight of the herbs, respectively. The filtrates were combined and concentrated using a pressure-reduced rotary evaporator at a temperature of 70 °C. The extract yield was 17.9%. The extracts were kept in a refrigerated desiccator and before each experiment, they were freshly dissolved in ddH2O to reach the desired concentrations.

The UHPLC instrument, including a Thermo Scientific Dionex Ultimate 3000 Series RS pump coupled with TCC-3000RS column compartments and a Thermo Fisher Scientific Ultimate 3000 Series WPS-3000RS autosampler controlled by Chromeleon 7.2 Software (Thermo Fisher Scientific, Waltham, MA, USA and Dionex Softron GmbH Part of Thermo Fisher Scientific, Germering, Germany), was used for analysis. Chromatographic separation was carried out at 30 °C on an Agilent poroshell 120 EC-C18 (3 mm × 100 mm, 2.7 µm). The mobile phase was delivered at a flow rate of 0.3 mL/min and consists of 0.1% formic acid-water and acetonitrile using a gradient elution as follows: 0–10 min with 5–17% B; 10–12 min with 17–17% B; 12–14 min with 17–22% B; 14–19 min with 22–22% B; 19–29 min with 22–32% B; and 29–30 min with 32–50% B; 30–34 min with 50–90% B. The full scanned data in negative was acquired at a resolving power of 70,000 FWHM at *m*/*z* 200. A scan range of *m*/*z* 100–1500 was chosen. The typical chromatogram is shown in Figure 1, while the characterization of chemical constituents of licorice aqueous extract by UHPLC-Q Extractive Orbitrap-HRMS is presented in Table 1.

For HPLC quantification, HPLC was performed on an Agilent HPLC 1260 system (Santa Clara, CA, USA) equipped with an auto-sampler unit, diode array detector (DAD) and a Agela venusil MP-C18 column (4.6 mm × 250 mm, 5 μm). The injection volume was 20 μL. The mobile phase was delivered at a flow rate of 1 mL/min and consists of 0.1% formic acid–water and acetonitrile using a gradient elution as follows: 0–25 min with 20–30% B; 25–35 min with 30–35% B; 35–50 min with 35–65% B; 50–60 min with 65–65% B; and 60–65 min with 20–65% B. Chromatograms were recorded at 230 nm. The column temperature was 30 °C. The following standards (Chunqiu Bio-Technology Co., Ltd., Purity: HPLC ≥ 98%) were examined: liquiritin, isoliquiritin, liquiritigenin and glycyrrhetinic acid. The data are shown in Table 2 and the values were expressed as mean ± SD (*n* = 6, 6 batches of preparations). Glycyrrhetinic acid and liquiritin were identified as the major compounds, representing 21.60 mg/g and 11.82 mg/g. The analysis was repeated consecutively using the same sample 6 times to determine precision. Additionally, the sample was analyzed at 0, 2, 4, 6, 12 and 36 h in stability tests. Six samples of licorice aqueous extract from the same batch were analyzed to measure the repeatability of this method. Relative standard deviations (RSD) of precision, stability and repeatability are shown in the following Table 5, indicating good precision, stability and repeatability of the method.

### 4.3. Animals

Non-pregnant Female Imprinting Control Region (ICR) mice weighing 18–22 g (6–8 weeks old) were obtained from the Experimental Animal Center of Yangzhou University. All animals were housed in a temperature of 25 °C, humidity of 50% and light controlled (12 h light/12 h darkness) vivarium with food and water ad libitum. The animal use protocol was approved by the animal ethics committee of the School of Chinese Material Medica at China Pharmaceutical University (Approval number: SCXK (Su) 2012-0004).

### 4.4. Isolation of Uterus and Measurement of Uterine Contraction

Female ICR mice were pretreated with estradiol benzoate (1 mg/kg/day) by intraperitoneal injection for 3 consecutive days before the ex vivo experiments. After the animals were sacrificed by cervical dislocation, the uteri were collected to a container filled with Locke’s solution (in mmol/L: NaCl 120, KCl 4.6, CaCl_2_ 1.5, MgSO_4_ 1.2, KH_2_PO_4_ 1.0, NaHCO_3_ 25 and glucose 11). After removal of the adherent fat tissue and mesenteric attachments of uterine strips, the isolated uteri were individually transferred and incubated in 20 mL of Locke’s solution in organ baths at 37 °C bubbled with 95% O_2_ and 5% CO_2_. Following this, the uterus was preloaded with 1 g tension, before the amplitude of uterine contraction became stable after equilibration for 45 min. Uterine contractions were monitored by a force-displacement transducer connected to a polygraph (Model BL420E+, Tai Meng, Chengdu, China). To evaluate the influence of licorice aqueous extracts on oxytocin-stimulated uterine contractions, oxytocin (0.01 U/mL) was added to the organ bath at 15 min before different concentrations of licorice and nifedipine (positive control) were cumulatively added. The contraction was monitored for about 10 min for each concentration. The data were presented as contraction amplitude and frequency.

### 4.5. Effects of Oxytocin-Stimulation on Isolated Uterus Ex Vivo

The isolated uteri were randomly divided into six groups and equilibrated for 45 min in Locke’s solution at 37 °C bubbled with 95% O_2_ and 5% CO_2_. The oxytocin (0.01 U/mL) was added to the incubation solution at six time points in different groups and allowed to react. After the reaction, the uteri tissues were collected for further investigation.

### 4.6. Effects of Licorice Aqueous Extract on Oxytocin-Stimulated Uterus Ex Vivo

The isolated uteri were randomly divided into six groups and equilibrated for 45 min in Locke’s solution at 37 °C bubbled with 95% O_2_ and 5% CO_2_. Each uterus of the treatment groups was stimulated by oxytocin (0.01 U/mL) and a combination of licorice (0.1, 0.2 and 0.4 mg/mL) or nifedipine (0.5 ng/mL) was added. The equal volume of DMSO was added into the control and oxytocin-stimulated model group. All groups were allowed to react for 60 min. After the reaction, the uteri tissues were collected for further investigation.

### 4.7. Histological Tests

The uterine tissues were fixed in 10% buffered formalin and dehydrated with a graded ethanol series and embedded in paraffin. The tissues were subsequently sliced into 5-μm thick sections using a histotome. The morphological evaluation of tissues stained with hematoxylin and eosin (H & E) was performed under a light microscope (OLYMPUS DX45, Tokyo, Japan). Identification standards for evaluating the contractile status of smooth muscle cells in the uterine inner annular layer based on morphological characteristics were ascertained. The short rod-shaped cells with shorter nucleus, defined as oval nucleated cells, were in contractile status after oxytocin-stimulation. However, the normal myometrial cells were slender with round nucleus, defined as fusiform nucleated cells.

### 4.8. Immunofluorescence

The same tissue sections prepared from the Method 2.7 were mounted on polylysine-coated slides and deparaffinized in dimethylbenzene solution three times once for 10 min. This was followed by progressively hydrated with successive washes at room temperature in 100% ethanol, 95% ethanol in PBS, 70% ethanol in PBS and PBS. After permeabilization for 15 min in boiling citrate solution (0.1% sodium citrate, 0.1% Triton X-100 in water), the tissue slides were washed twice with PBS at room temperature. Non-specific binding sites were blocked with 3% BSA in PBS for 1 h, following which the anti-HSP27 and anti-α-SMA primary antibodies (1:100 in blocking buffer) were incubated with tissue slides overnight at 4 °C. The slides were washed three times with PBS, before the bound antibody was detected using Alexa Fluor^®^ 647 conjugated anti-rabbit and Alexa Fluor^®^ 488 conjugated anti-mouse secondary antibodies diluted 1:200 for 1 h at room temperature in a black wet box protected from light. The slides were washed three times with PBS, before being counterstained with DAPI dye followed by three rinses with water. The tissues were covered with anti-fade mounting medium and observed under a fluorescence microscope (DM2500, Leica Microsystems, Wetzlar, Germany). In the negative control, the primary antibody was instead replaced with PBS.

### 4.9. Western Blot Analysis

Uterine tissue samples were prepared by homogenization in modified radioimmunoprecipitation assay buffer (RIPA buffer: 1× PBS, 1% Igepal CA-630, 0.5% sodium deoxycholate, 0.1% SDS, 10 mg/mL PMSF, 30 μL/mL aprotinin and 100 mM sodium orthovanadate), maintained in constant agitation for 1.5 h at 4 °C and subsequently centrifuged twice at 12,000× *g* for 5 min at 4 °C. The supernatants were collected and the protein concentration was determined by the BCA kit. A total of 40 µg of total protein was applied to each well of a 10% SDS polyacrylamide gel and submitted to electrophoresis for 2 h at 120 V with a set of reference protein markers. Following this, the proteins were transferred onto PVDF membranes (Millipore Corp, Bedford, MA, USA) with 200 mA for 1.5 h at room temperature using a transfer buffer composed of 25 mmol/L Tris base, 192 mmol/L glycine and 20% methanol. The blots were blocked for 1 h at room temperature with a blocking buffer of 10 mmol/L Tris at a pH of 7.5, 100 mmol/L NaCl and 0.1% Tween 20. The blocking buffer was decanted and the blots were incubated with anti-HSP27, anti-pHSP27-Ser15 and anti-α-SMA primary antibodies. After washing three times with a TBS buffer composed of 10 mmol/L Tris, pH 7.5, 100 mmol/L NaCl and 0.1% Tween 20, the membrane was incubated with a peroxidase-conjugated secondary antibody depending on the source of primary antibody. The blots were finally detected with enhanced chemiluminescence reagent (ECL) and visualized using the ImageLab software 4.1 (Bio-Rad, Hercules, CA, USA). Each experiment was repeated three times in order to ensure reproducible results.

### 4.10. ChemGPS-NP Analysis

The principal component analysis (PCA) based global chemical space map ChemGPS-NP (http://chemgps.bmc.uu.se) is an online tool for exploration of the regions of natural products with biologically relevant chemical space. It consists of eight principal components (dimensions, PC), derived from 35 molecular descriptors describing the physical-chemical properties. The basic interpretation of the eight dimensions of ChemGPS-NP are presented in Table 6. The ChemGPS-NP descriptors were calculated for seven main chemical ingredients from licorice aqueous extracts (Figure 1 and Table 1, Compd. **1**–**7**) based on their structure information as simplified molecular input line entry specification (SMILES). Following this, compounds were mapped in the ChemGPS-NP global map using interpolation in terms of PCA score prediction together with a reference set of known inhibitors, targeting p38, MK2 and PKCδ separately. The source of known inhibitors for comparison was collected from online ChEMBL database and a cutoff of 10 μM in IC_50_ was performed. The coordinates were plotted using SigmaPlot software (SigmaPlot 12.5, San Jose, CA, USA).

### 4.11. Molecular Docking

The protein crystal structures used in the docking studies were obtained from the protein data bank (PDB ID: 3R2Y for MK2; PDB ID: 1XJD for catalytic domain of PKC). The internal known inhibitors in 3R2Y and 1XJD were removed and extracted from the protein structures, re-docked into the active site to verify the docking method’s reliability (RMSD is 1.26 Å and 0.89 Å respectively, which shows the reliability of docking method). The tested compounds of SMILES format were converted to three-dimensional (3D) structures of PDB format using online SMILES translator and structure file generator (http://cactus.nci.nih.gov/translate). Molecular docking was performed using software of Autodock4.0 (AutoDock4 and AutoDockTools4, Scripps Research Institute, MB-5 10550 N. Torrey Pines Rd., La Jolla, CA, USA). The size of the grid boxes was 40 × 40 × 40, whose center coordination were 6.502, 18.269 and 45.549 (MK2) as well as 56.374, 8.021 and 3.610 (PKC) with the spacing of 0.375 Å. The default values were used for all other parameters. The predicted protein–ligand complexes were optimized and ranked according to the empirical scoring function, which estimates the binding free energy of the ligand receptor complex.

### 4.12. Statistical Analysis

Results were presented as means ± SEM. Data were analyzed using GraphPad Prism version 5 (GraphPad Software, San Diego, CA, USA) by one-way ANOVA followed by Tukey’s Honestly Significant Difference test for multiple comparison testing to estimate the significance of the results. A value of *p* < 0.05 was considered statistically significant, while *p* < 0.01 was considered statistically highly significant.

## Figures and Tables

**Table 1:** Characterization of chemical constituents of licorice aqueous extract by UHPLC-Q Extractive Orbitrap-MS.

Compounds	tR	Identification	Molecular Formula	[M − H]^−^	MS/MS (*m*/*z*)
**1**	14.82	Liquiritin apioside or isomer	C_26_H_30_O_13_	549.16046 (0.351)	255, 153, 135, 119, 91
**2**	15.26	Liquiritin *	C_21_H_22_O_9_	417.1191 (2.784)	255, 153, 135, 119
**3**	20.85	Isoliquiritin apioside	C_26_H_30_O_13_	549.16150 (2.245)	255, 153, 135, 119, 91
**4**	22.16	Isoliquiritin *	C_21_H_22_O_9_	417.11899 (0.881)	255, 153, 135, 119, 91
**5**	24.23	Liquiritigenin *	C_15_H_12_O_4_	255.06618 (3.9)	153, 135, 119, 91
**6**	32.79	Isoliquiritigenin *	C_15_H_12_O_4_	255.06635 (4.566)	237, 153, 119, 91
**7**	32.81	Glycyrrhetinic acid *	C_42_H_62_O_16_	821.39648 (1.3)	759, 469, 351

* Compared with a reference standard.

**Table 2:** Quantification of compounds in licorice aqueous extract by HPLC.

Compounds	LOD (μg/mL)	LOQ (μg/mL)	Quantity (mg/g)
Liquiritin	0.678	2.104	11.82 ± 0.17
Isoliquiritin	1.333	3.333	1.28 ± 0.06
Liquiritigenin	0.567	1.700	0.12 ± 0.01
Glycyrrhetinic acid	6.217	12.433	21.60 ± 0.60

NB: LOD: limit of detection; LOQ: limit of quantification.

**Table 3:** Interaction of MK2 protein (PDB: 3R2Y) with liquiritin, isoliquiritin, liquiritigenin and isoliquiritigenin using the molecular docking software AutoDock 4.0.

Compound	Binding Energy (kcal/mol)	Number of Hydrogen Bonds	Hydrogen Bonding	Inhibition Constant, Ki (μM)
Liquiritin	−7.65	3	LEU141, ASP207, LEU72	2.47
Isoliquiritin	−6.69	2	LEU141, ASP207	12.45
Liquiritigenin	−6.97	4	LYS93, LEU141, LEU72, GLU139	7.83
Isoliquiritigenin	−6.74	4	LEU141, GLU139, GLU145, ASN191	11.40

**Table 4:** Interaction of catalytic domain of PKC (PDB: 1XJD) with liquiritigenin and isoliquiritigenin using molecular docking software AutoDock 4.0.

Compound	Binding Energy (kcal/mol)	Number of Hydrogen Bonds	Hydrogen Bonding	Inhibition Constant, Ki (μM)
Liquiritigenin	−7.15	3	LEU461, LYS409	5.73
Isoliquiritigenin	−6.61	4	LEU461, GLU426, LYS409	14.16

**Table 5:** Validation of HPLC analysis method in terms of precision, stability and repeatability.

Compounds	Precision (*n* = 6) RSD (%)	Stability (*n* = 6) RSD (%)	Repeatability (*n* = 6) RSD (%)
Liquiritin	1.13	0.64	1.38
Isoliquiritin	0.67	0.83	2.21
Liquiritigenin	1.72	2.24	1.62
Glycyrrhetinic acid	1.07	0.74	2.37

**Table 6:** Summary of the most important contributing characteristics of the eight dimensions in ChemGPS-NP.

PC	Physical-Chemical Properties
1	Size, shape, polarizability
2	Aromaticity, conjugation-related properties
3	Lipophilicity, polarity, H-bond capacity
4	Flexibility, rigidity
5	Electronegativity, number of nitrogens, halogens, amides
6	Number of rings, rotatable bonds, amides, hydroxyl groups
7	Number of double bonds, oxygens, nitrogens
8	Aromatic and aliphatic hydroxyl groups, unsaturation, LAI (Lipinsky Alert Index) *

* Drug-like indices.

## Abbreviations

PD	primary dysmenorrhea
HSP27	heat shock protein 27
PCA	principal component analysis
PKC	protein kinase C
p38 MAPK	p38 mitogen-activated protein kinases
MK2	MAP kinase-activated protein kinase 2
